# A method to determine the correct photocatalyst concentration for photooxidation reactions conducted in continuous flow reactors

**DOI:** 10.3762/bjoc.16.78

**Published:** 2020-04-27

**Authors:** Clemens R Horn, Sylvain Gremetz

**Affiliations:** 1 Corning European Technology Center, 7 Bis Avenue de Valvins, F-77215 Avon Cedex, France

**Keywords:** catalyst concentration, channel height, flow chemistry, photooxidation

## Abstract

When conducting a photooxidation reaction, the key question is what is the best amount of photocatalyst to be used in the reaction? This work demonstrates a fast and simple method to calculate a reliable concentration of the photocatalyst that will ensure an efficient reaction. The determination is based on shifting the calculation away from the concentration of the compound to be oxidized to utilizing the limitations on the total light dose that can be delivered to the catalyst. These limitations are defined by the photoflow setup, specifically the channel height and the emission peak of the light source. This method was tested and shown to work well for three catalysts with different absorption properties through using LEDs with emission maxima close to the absorption maximum of each catalyst.

## Introduction

The renaissance of photochemistry that occurred over the last decade has been described as “old light through new windows”, and much of this work centered on the combination of light with continuous flow reactors [1–6]. The reason for this increased interest is that continuous flow photochemical reactors can overcome various limitations that occur when the same reaction run in batch reactors [7]. For example, the flow reactor can overcome the difficulties that occur upon scale-up. The most desirable approach for industrial-scale reactions is described by the quote “the best approach to the scale-up of a photoreaction seems to be the enlarging of a laboratory-type prototype reactor, with the important parameters being changed as little as possible” [8]. This phrase describes the approach used to scale up flow reactors to an industrial scale [9–10]. This is particularly true when it comes to biphasic reactions, like a gas/liquid reaction as is the case for photooxidations [11]. An interesting aspect of photooxidations is that the combination of light, the “greenest reagent” oxygen, and a catalyst as well as applying mild process conditions under process-intensified conditions can be considered a very sustainable setup [12–14].

The scaling-up of photoflow reactors has also been affected by the increased use of LED sources over mercury arcs and other light sources commonly used in industry and classical synthetic organic photochemistry. LEDs typically have much narrower emission spectra when compared to these light sources, all of which have broad emission spectra except for low-pressure sodium vapor lamps [15–16]. These broad emission spectra pose some further complications as they are ultimately a rather inefficient way to use the energy and provide wavelengths that can effectively increase the risk of side reactions [17]. The fact that only a portion of the emitted light is used in the reaction can further complicate the scale-up of the light source and design of the reactor. LED sources have continuously been evolving and will continue to do so because when being employed, the light source is no longer a limiting factor in the design of a larger-scale photoreactor setup [18]. Since an LED provides a narrower emission wavelength, the appropriate wavelength above 365 nm can be selected for every reaction, and the scale-up can be achieved by simply increasing the number of the LEDs applied [19]. The narrower spectra, while providing many benefits and likely reducing the occurrence of side reactions, also facilitate that other factors become more important. Notably, an exact description of the photoflow setup is now crucial to ensure reproducible experiments [20–21].

## Results and Discussion

### The effect of 1 mol % photocatalyst

**Figure 1:**
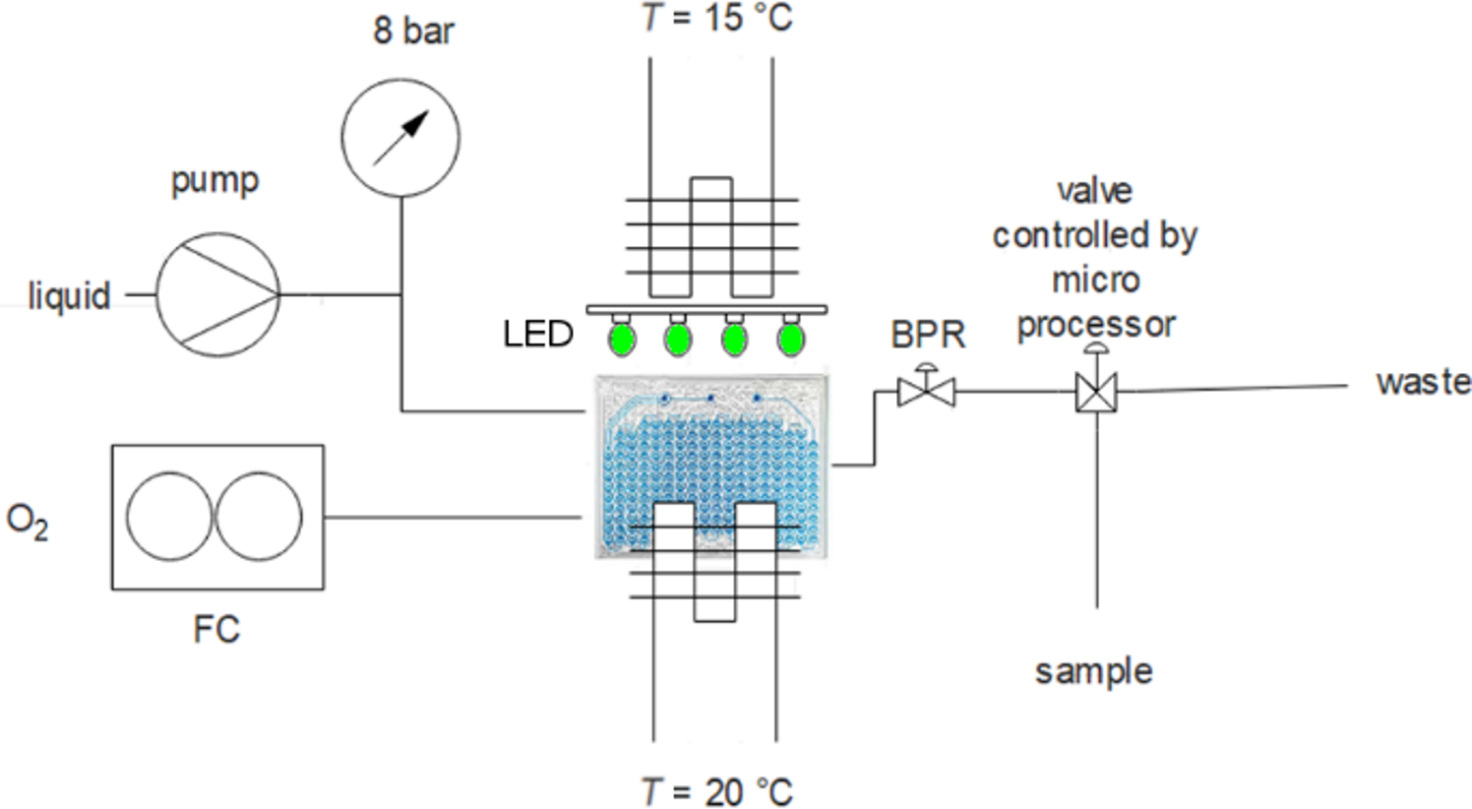
Reaction setup (FC: flow controller, BPR: back pressure regulator).

**Scheme 1:**
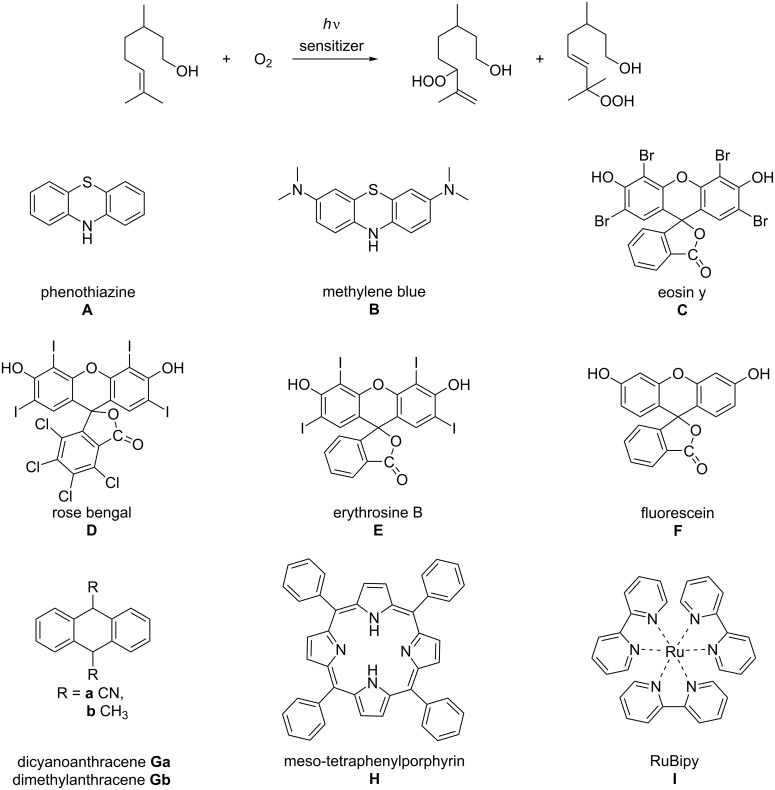
Photocatalysts **A**–**I** screened for the oxidation of citronellol.

This work was accomplished using the Corning^®^ Advanced-Flow^tm^ Lab Photo Reactor, providing a robust platform for a rapid screening of the reaction conditions (e.g., the wavelength) and, for photooxidation reactions, the catalysts. For a schematic reaction setup see Figure 1, and further details can be found in Supporting Information File 1 [22]. As a test reaction, we rapidly screened 9 photocatalysts for the oxidation of citronellol (Scheme 1), using the conditions described by Lévesque and Seeberger [23].

**Figure 2:**
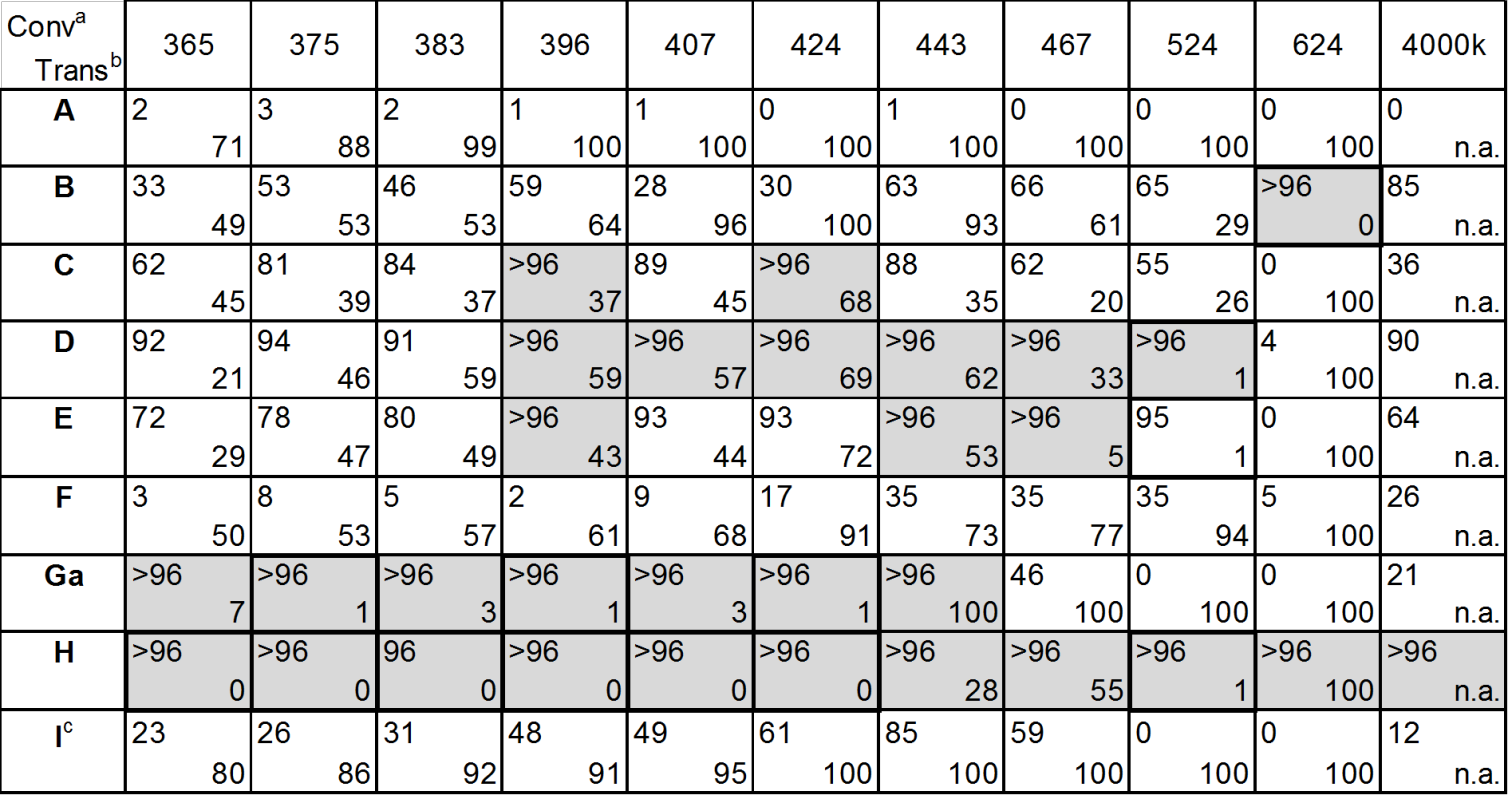
Conversion and transmission at fixed reaction conditions (0.5 N citronellol, 1 mL/min,1 mol % catalyst, 2 equiv oxygen, 8 bar, 20 °C, reactor volume: 2.7 mL, solvent: ethanol (**B**, **C**, **D**, **E**, and **F**) or dichloromethane (**A**, **G**, **H**, and **I**)). ^a^The conversion was determined by GC after quenching with PPh_3_. ^b^Transmission (in %) of the starting solution at 0.5 mm pathlength. ^c^Catalyst concentration: 0.1 mol %.

The reaction conditions used for the experiments shown in Scheme 1 are detailed in Figure 2 below, and the best results are highlighted in grey.

The conversions of the photooxidation reactions were determined under similar reaction conditions using 11 different wavelengths (10 sources with defined wavelength and 1 white light source with a color temperature of 4000 K). At the same time, the transmission spectra of all starting solutions were recorded using a cuvette with a path length of 0.5 mm, which is close to that of the fluidic module in the reactor (0.4 mm). The lower-right value in each field of Figure 2 corresponds to the transmission recorded at the LED emission maximum. In about 30% of the tests, a complete conversion was obtained (highlighted in grey in Figure 2), but only in 15% of the cases, the catalyst concentration was high enough to absorb all the light at the given wavelength (highlighted with a bold frame, transmission < 1%).

**Figure 3:**
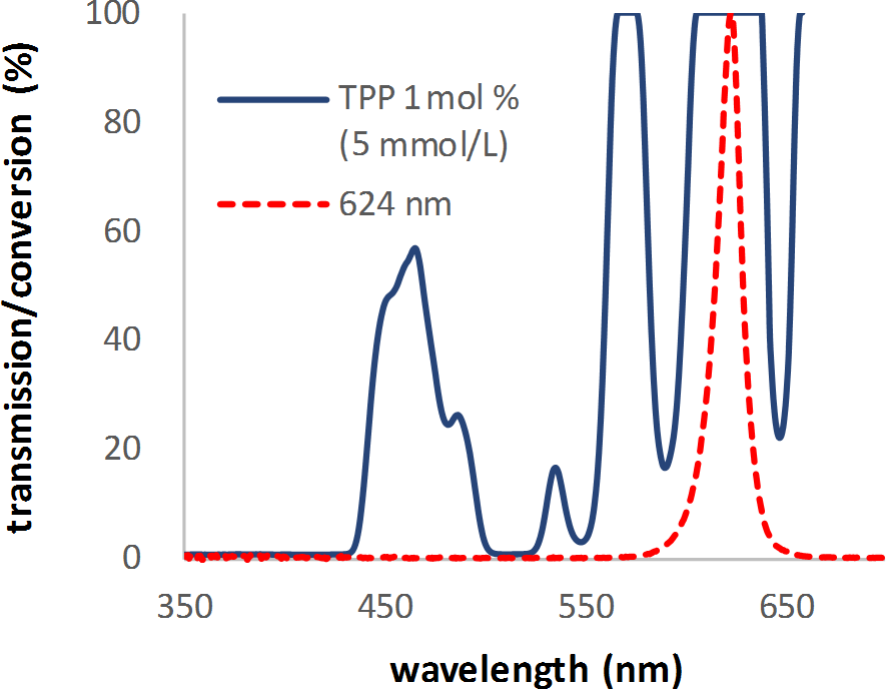
Measured transmission spectrum of a 5 mmol/L (1 mol %) solution of TPP (**H**) in dichloromethane with 0.5 N citronellol and the emission spectrum of the LED with an emission maximum at 624 nm.

Overall, the results show that 1 mol % of the catalyst was not the ideal concentration for the various initiators. For example, in the extreme case of using tetraphenylporphyrin (TPP, **H**), the concentration was so high that a complete conversion was obtained at any wavelength. This included a case where the reaction was performed at 624 nm, with the measured transmission for the solution being 100% (Figure 3). There was still enough overlap with the Q bands of **H** at 593 and 649 nm to provide a high enough light intensity for the reaction, and thus causing the complete conversion.

### A method to obtain an estimation of the required photocatalyst

As the photooxidation reaction has been shown to be dependent on the photocatalyst concentration, a simple method to determine the required amount in a reaction mixture is necessary. Further, when considering an industrial process, the concentration of the photocatalyst will have not only an economic impact but could also impact the downstream process or subsequent synthetic steps. It may be noted that in typical studies on photooxidation reactions, the concentration of the reactants is usually varied, while the catalyst concentration is rarely addressed [24–26].

To study the required photocatalyst concentration, we pursued a different approach based on the realization that in a photochemical reaction, the often overlooked key reagent is, in fact, the light, and through the subsequent interaction between light and the photocatalyst, the light is transformed. Following this logic led to the observation that one needs to more carefully consider not only the concentration of the catalyst but the details of how and to what extent the light interacts with the catalyst in the region where it is exposed to the light. A good starting point for this analysis was the Bunsen–Roscoe law of reciprocity, which states that a photochemical effect is directly proportional to the total energy dose, which, for a flow reactor, is the product of the light intensity and time [27–29]. The light intensity can be considered as the limiting factor in a flow setup as the space around the reaction channel is limited, as is the power of the light source. The exposure time has less limitations as it is controlled by the flow rate. This implies that in order to conduct an efficient reaction, the use of the light has to be optimized such that the quantity of light reaching the reaction mixture in the channel is large enough to be totally absorbed.

Our approach to considering light more conspicuously as a reagent uses the Bouguer–Lambert–Beer law as a starting point to estimate a concentration close to the optimum. The law in its basic definition is not entirely applicable to a flow reactor but will nevertheless provide a convenient starting point [30–31]. The analysis of the required concentration of the catalyst begins with recognizing how the law is applied and what the differences from the batch approach that it describes are [32]. A flow reactor has a channel height that is a well-defined rectangle, allowing it to be used synonymously to the path length of the light. However, since the path length of the channel is much shorter than the path length used in a classical cuvette measurement, the values obtained from the Beer’s law plot may be less accurate. A second contribution to the approximation is that to use all the light, the transmission through the channel needs to be below 1%, which corresponds to an absorption of >2. This condition also limits the use of the Bouguer–Lambert–Beer law since at these high absorption values, the extinction coefficient may exhibit a nonlinear behavior with respect to the concentration of the catalyst [33]. Taking note of the facts above but pressing on and applying Beer’s law using a path length fixed at 0.04 cm and an extinction of 2, the molar extinction coefficient still needs to be calculated. Therefore, the molar extinction coefficient needs to be measured separately in a UV–vis spectrometer as the emission maximum of the LED source does, in most cases, not correspond to the absorption maximum of the catalyst. Furthermore, the spectra need to be obtained under the same conditions under which the reactions take place, i.e., in the same reaction medium, at the same temperature and, if possible, at the same path length. The first two parameters are extremely important as the absorption peak will shift in both height and wavelength depending on the reaction medium and temperature [34]. The path length should be as close as practical to that of the reactor as this will reduce the error imparted by the limitations of the Bouguer–Lambert–Beer law. Table 1 shows the values measured for the extinction coefficient, the LED peak, and the absorption maximum based on the measured values and the predicted concentration for the catalyst using the channel height of 0.04 cm.

**Table 1:** Measured extinction coefficients and estimated ideal catalyst concentrations.

sensitizer	wavelength [nm]	extinction coefficient [L/(mol·cm)]	literature reference	calculated sensitizer concentration [mmol/L]

rose bengal (**D**)	**560**	**90000**	**90400** [35]	**0.56**
	524^a^	37400		1.34
	524^b^	38100		1.31
TPP (**H**)	424	349000		0.20
	**419**	**401000**	**443000** [35]	**0.12**
	407	158000		0.32
DMA (**Gb**)	407	2800		17.86
	**398**	**7300**	**7500** [36]	**6.85**
	396	6000		8.33
	383	6900		7.25
	375	6500		7.69
	365	3800		13.16

Rose bengal (**D**) was measured in ^a^ethanol or ^b^acetonitrile, with 0.5 N citronellol; TPP (**H**) was measured in toluene with 1 N alpha-terpinene; DMA (**Gb**) was measured in toluene with 0.5 N alpha-terpinene.

**Figure 4:**
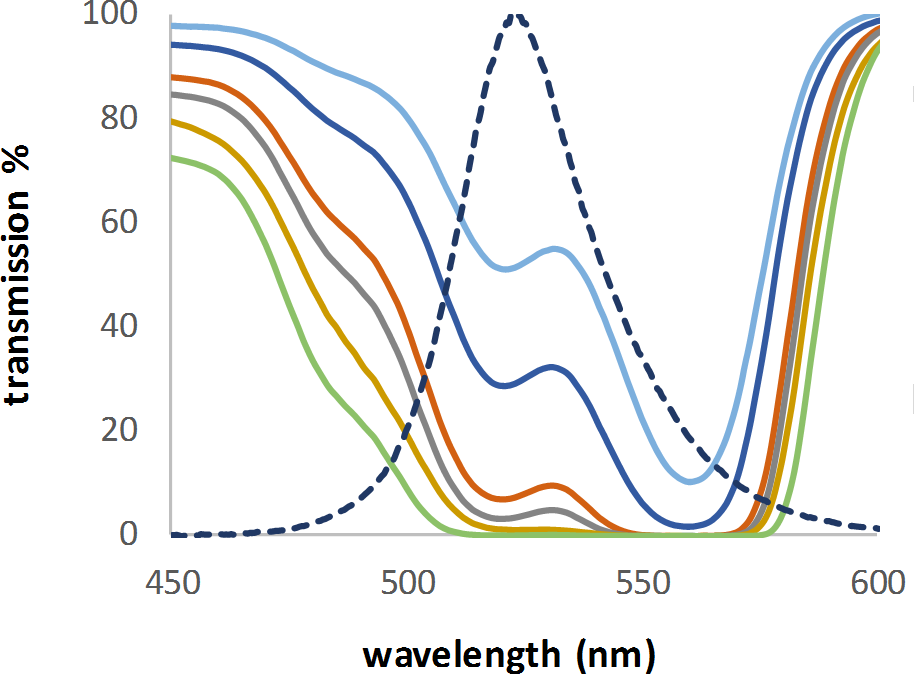
Transmission spectra of rose bengal (**D**) and the emission spectrum of an LED with a maximum at 524 nm (scaled to 100%).

**Figure 5:**
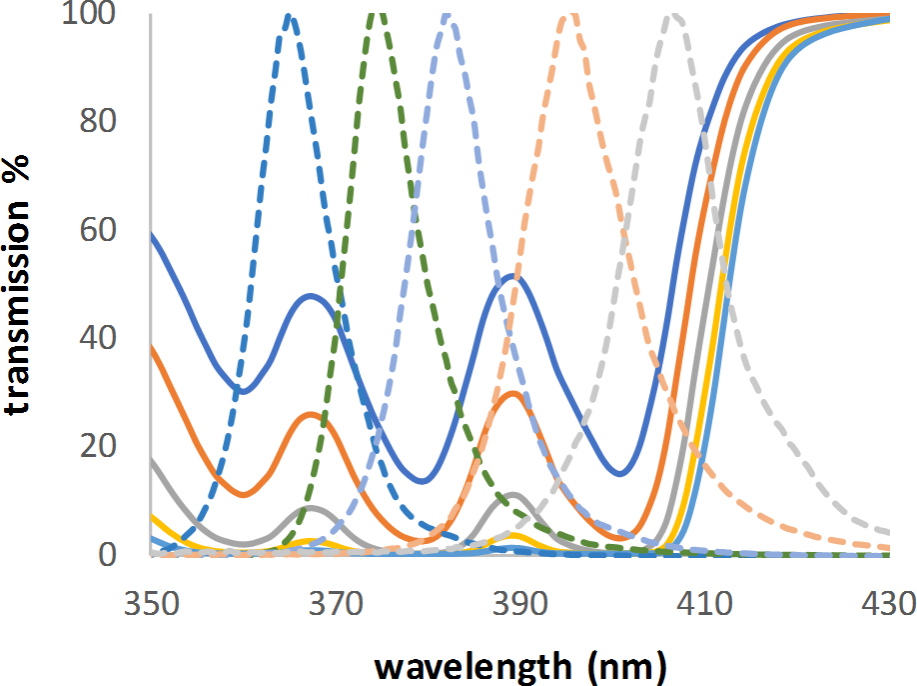
Transmission spectra of dimethylanthracene (**Gb**) and emission spectra of LEDs with maxima at 365, 375, 383, 394, and 407 nm, respectively (all scaled to 100%).

**Figure 6:**
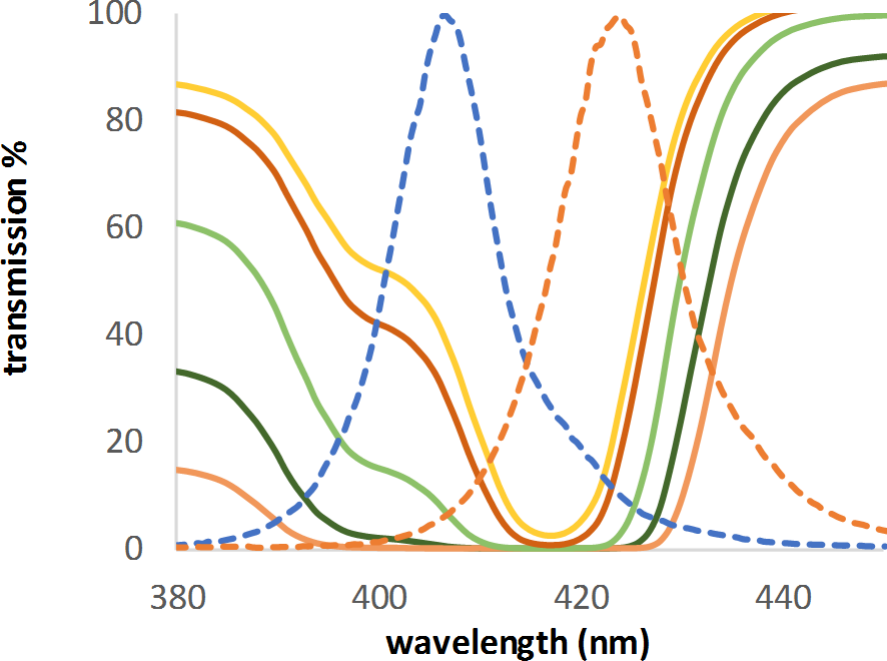
Transmission spectra of TPP (**H**) and emission spectra of LEDs with maxima at 407 and 424 nm, respectively (both scaled to 100%).

Figures 4–6 serve to illustrate three possible examples. For rose bengal (**D**, Figure 4), the emission maximum of the used LED fits nearly to a secondary peak. Dimethylanthracene (DMA, **Gb**, Figure 5), with three absorption peaks, covers a wide range of possible LEDs. TPP (**H**, Figure 6) is the opposite of DMA (**Gb**). It has a very high absorption in a very narrow peak range (that is, a Soret band). As can be seen in Figure 6, the absorption maximum (here transmission 0%) is close to the emission spectra of two of the LEDs, with only 5 and 12 nm difference, respectively. Nevertheless, the calculated concentrations were nearly two or three times higher when compared to the lowest concentration, which would be required for a perfect fit between the absorption maximum and the LED emission. This highlights why it is so important to measure the absorption in the intended reaction medium. A solvent change could shift the maximum by a few nm (TPP: 416 nm in chloroform [37], 418 nm in benzene [38], and 419 nm in toluene [35]).

### Reactions using calculated photocatalyst concentrations

**Scheme 2:**
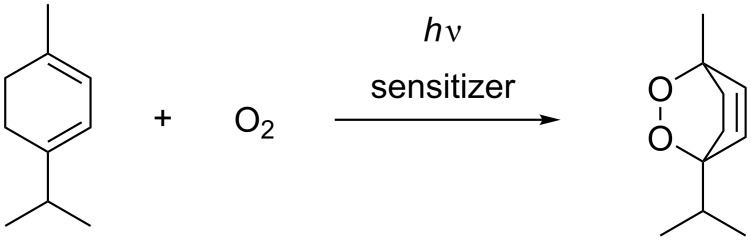
Photooxidation of alpha-terpinene.

**Figure 7:**
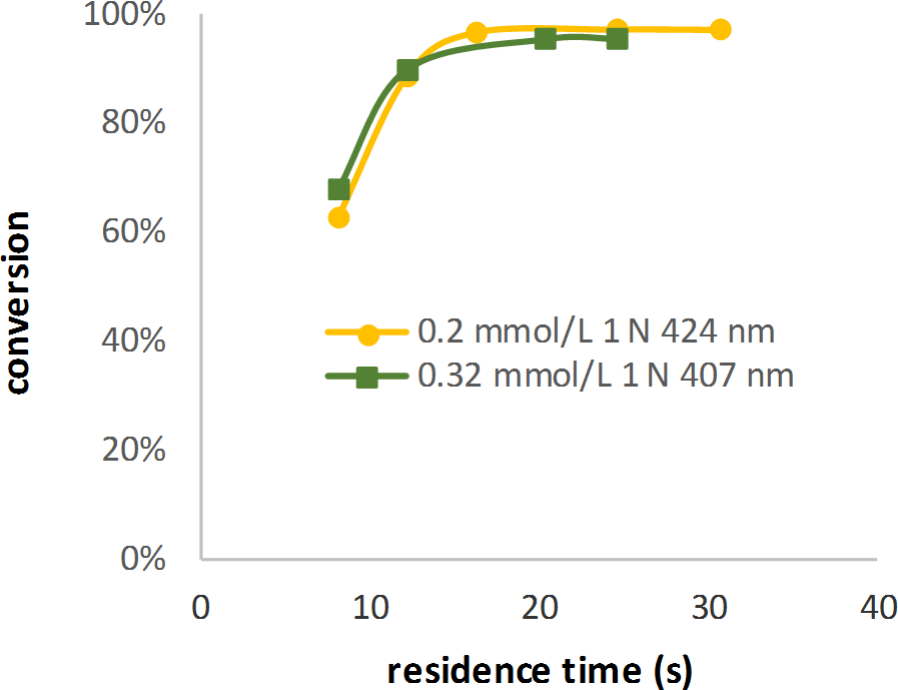
Conversion of alpha-terpinene using the wavelength-adapted TPP (**H**) concentrations.

The concentrations calculated for the catalyst were tested using the oxidation of alpha-terpinene (Scheme 2) and the oxidation of citronellol (Scheme 1). All reactions were conducted with 2 equiv of oxygen at 8 bar and 20 °C. The temperature of the light source was kept constant at 15 °C. It is very important to control the temperature of the reaction and the LEDs as the emission and absorption maxima are dependent on the temperature. The (minimum) exposure times were calculated using the liquid flow rate and the flow rate of the gas at the entrance. The results (Figure 7) show that similar results were obtained for the photooxidation of alpha-terpinene using TPP (**H**) at different wavelengths with the corresponding calculated amount of the catalyst.

**Figure 8:**
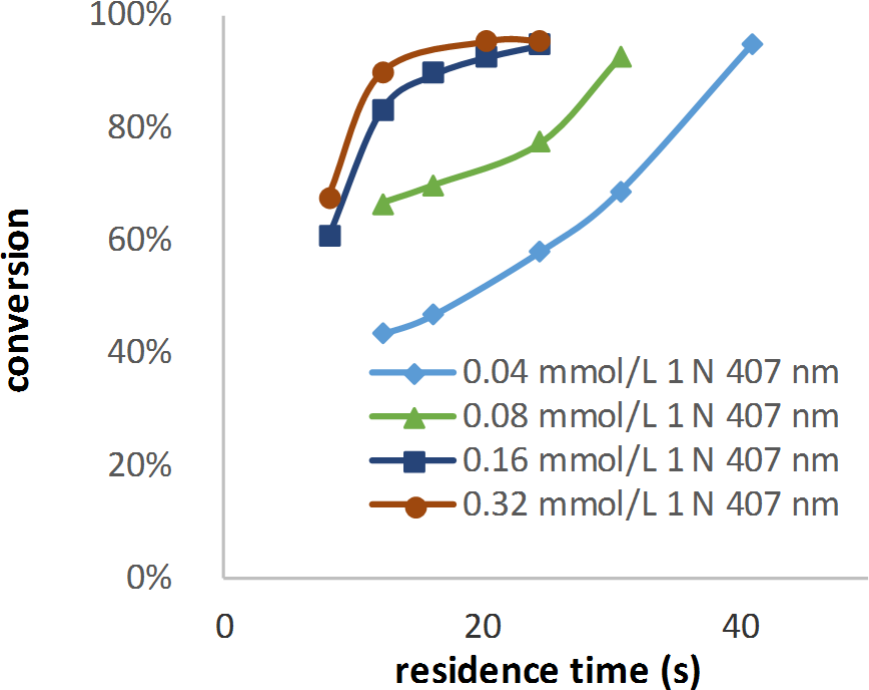
Conversion of alpha-terpinene at different TPP (**H**) concentrations.

The reduction of the catalyst concentration to half of the calculated value led to a small decrease in the conversion. This decrease became much larger when the catalyst concentration was further decreased by half again (Figure 8). This exponential decrease can be explained by the fact that the transmission of the light increased exponentially with a decreasing amount of the catalyst.

**Figure 9:**
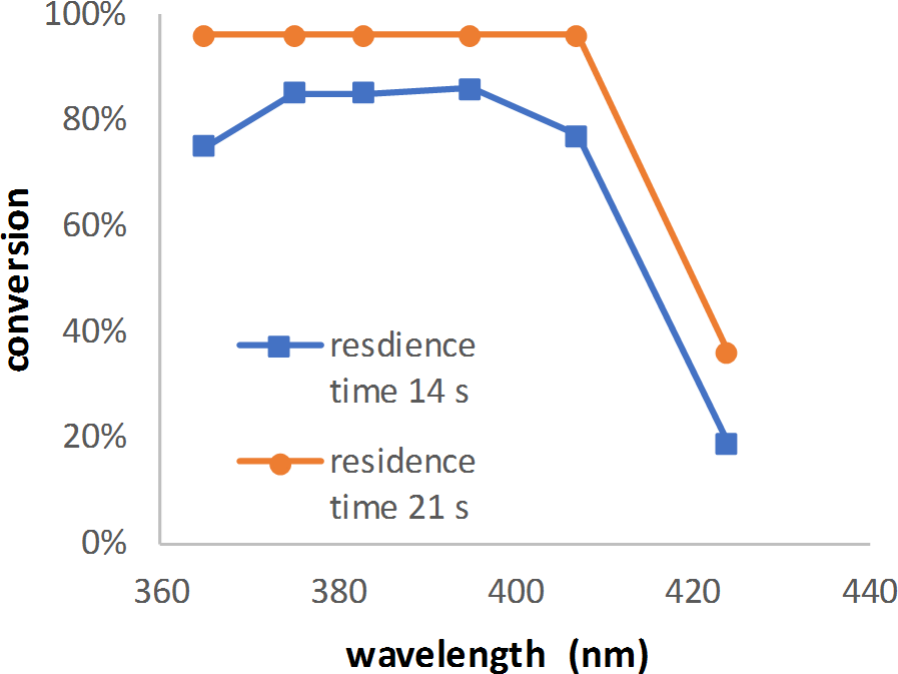
Conversion of alpha-terpinene (0.5 N) as a function of the wavelength using DMA (**Gb**) as the catalyst (7.25 mmol/L).

When DMA (**Gb**) was used and short exposure times were applied, the expected difference in conversion for the wavelengths 365 nm and 407 nm was seen: the reaction was not complete for any of the used wavelengths (Figure 9). This is completely in line with the observations made for TPP catalyst (**H**) concentrations.

**Figure 10:**
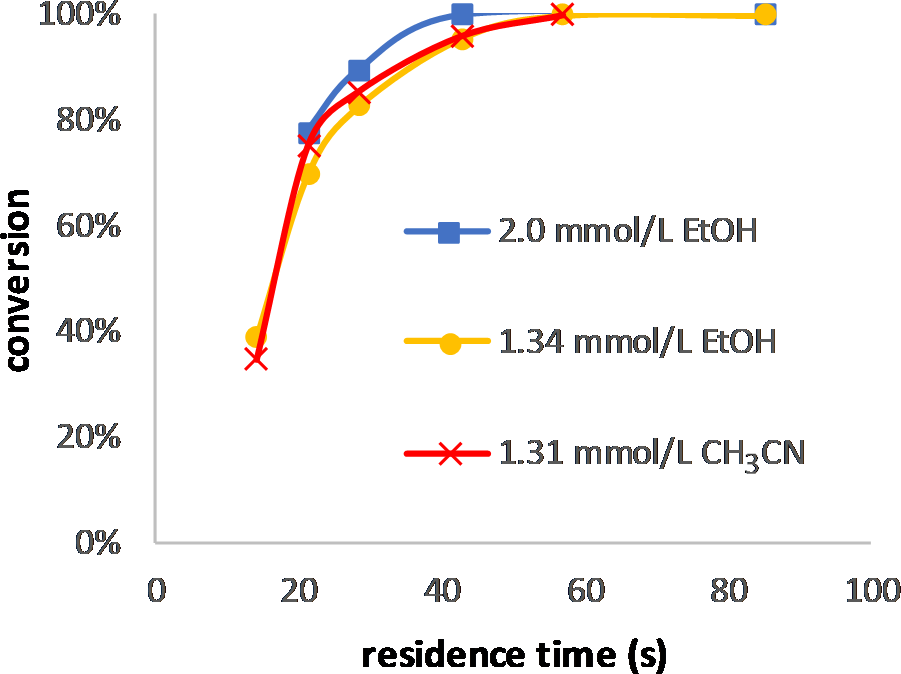
Conversion of citronellol at different concentrations of rose bengal (**D**).

Rose bengal (**D**) gave similar results: increasing the amount of the catalyst by 50% provided a slightly higher conversion for high flow rates, and the change of the solvent from ethanol to acetonitrile had the same impact (Figure 10). This can be attributed to the higher absorption of **D** in acetonitrile [39]. Those two results show the limitation of our approach because if the Bouguer–Lambert–Beer law would have been valid, the conversion should have been the same for the three conditions.

**Figure 11:**
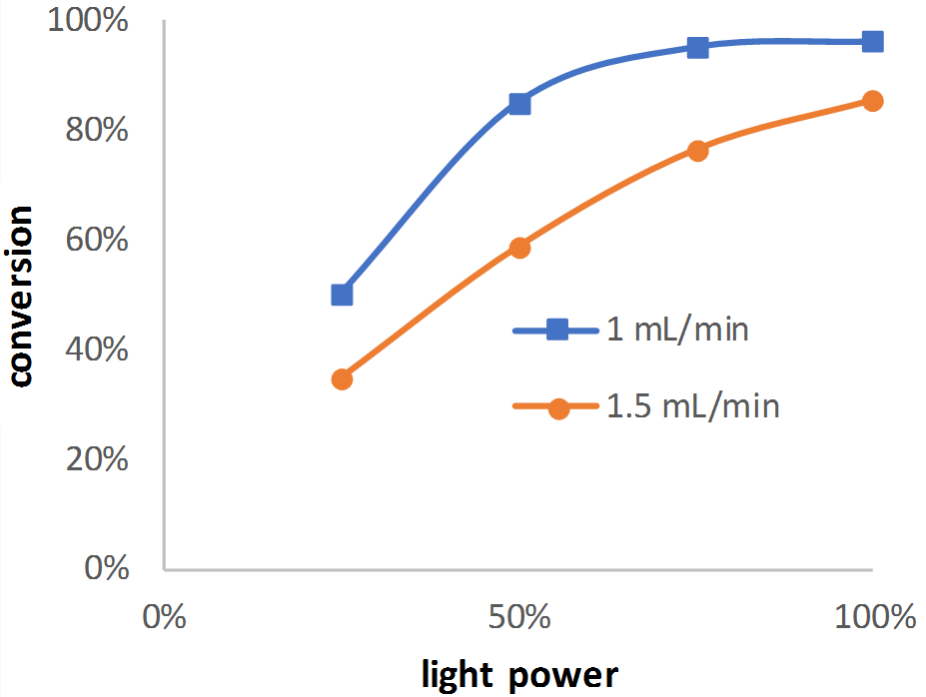
Conversion of citronellol as a function of the light power (0.5 mol/L of citronellol, 1.34 mmol/L rose bengal (**D**).

Our approach was to provide a simple means to optimize the catalyst used in conjunction with the maximum amount of light. As such, the question concerning the effect obtained upon variation of the light power arose. The answer to this can be found in Figure 11: rather large changes in the conversion of citronellol were observed as a function of the light power, and a greater effect was unsurprisingly observed for the slower flow rate.

**Figure 12:**
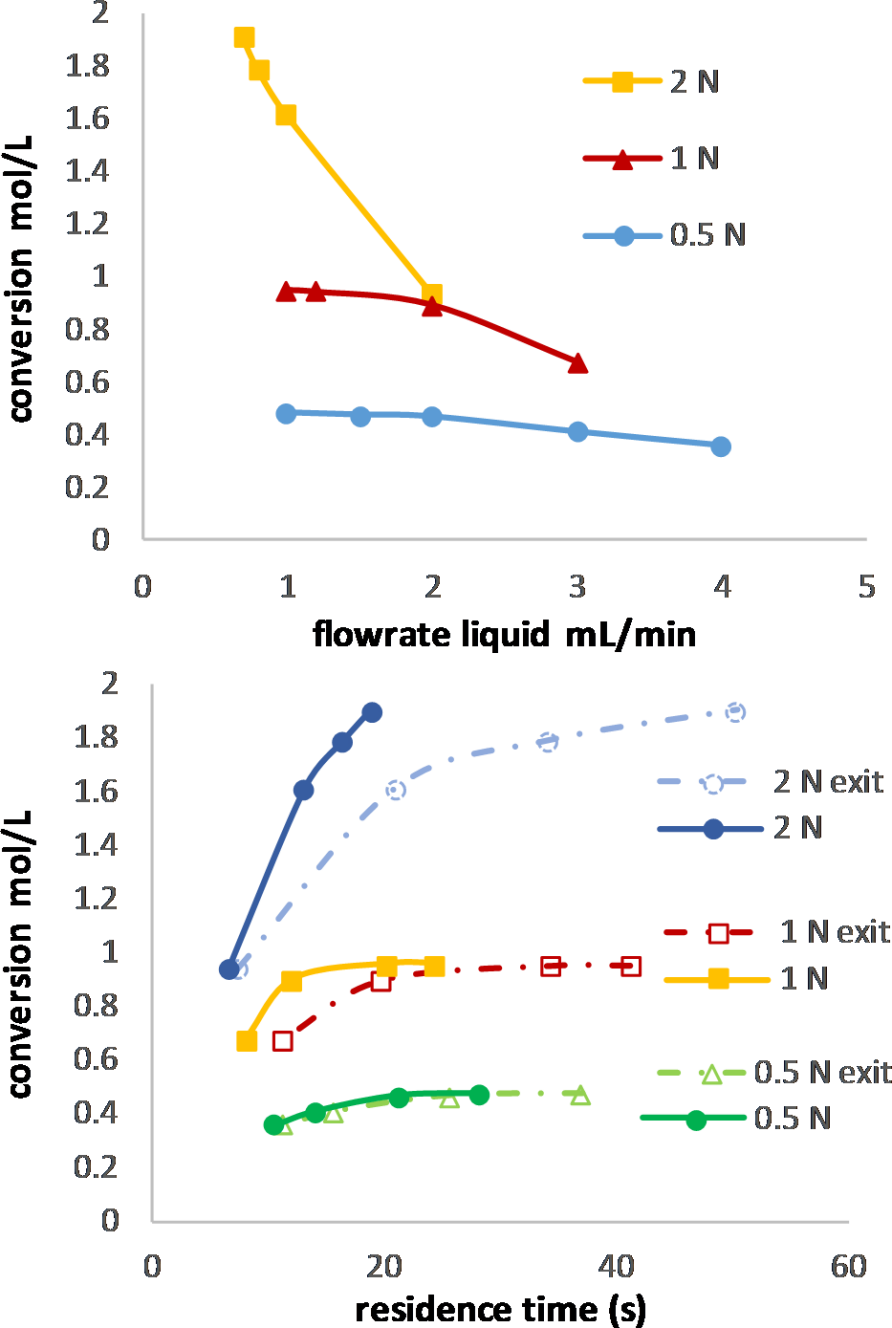
Absolute conversion of various concentrations of alpha-terpinene at 407 nm using 0.32 mmol/L of TPP (**H**) as a function of the liquid flow rate (top) and the residence time (bottom).

The effect of varying the concentration of the reactant concentration of alpha-terpinene while keeping the TPP (**H**) concentration constant (0.32 mmol/L) is shown in Figure 12.

Here, the ratio of alpha-terpinene to the catalyst decreased from 0.064 mol % to 0.016 mol %, and the conversion increased in a similar manner to that caused by the concentration changes. An exact comparison was not possible as it would be extremely difficult to calculate or even estimate the exact residence time. The residence times in Figure 12 were calculated based on the flow rate of the entrance and exit measurements (the conversion of the oxygen gas was considered). The reduction of the gas caused by the solubility of oxygen at the given pressure in the reaction mixture was also not considered, but for pure toluene, it would be 14% (0.5 N) or 4% (2 N) less gas volume [40]. Furthermore, the gas-to-liquid ratio change with the modification of the alpha-terpinene concentration impacts the flow pattern (droplet/bubble size), which could, in turn, have an impact on the conversion [41]. But the main limitation was the light itself, as can be seen in Figure 11. About the same absolute conversion was obtained for similar liquid flow rates independent from the residence time.

## Conclusion

We developed a reliable, quick, and simple approach to find the appropriate starting concentration of a photocatalyst for a photooxidation reaction in a flow reactor. This was achieved by linking the catalyst concentration to the channel height of the flow reactor and to the emission spectrum of the light source, using the Bouguer–Lambert–Beer law. Since the conditions applied were outside of, but close to the range of this law, the determined concentrations will be close to, but not exactly the optimum. The approach was tested with different light sources, and it was shown that it is important to match the LED to the reaction absorption spectrum. This is important as a variation of only a few nm can have a big impact on the catalyst concentration needed. We believe that this methodology will help in choosing the right catalyst and the right wavelength to eliminate scale-up issues encountered during the industrialization of photooxidation reactions.

## Supporting Information

File 1 Descriptions of material and methods, flow setup, and LED characterization.
