# Supplementary material for: A method to determine the correct photocatalyst concentration for photooxidation reactions conducted in continuous flow reactors
Source: Beilstein J Org Chem. 2020 Apr 27;16:871–9. doi: 10.3762/bjoc.16.78 (PMC7214865; doi:10.3762/bjoc.16.78)
Supplement: File 1 — Descriptions of material and methods, flow setup, and LED characterization. [file Beilstein_J_Org_Chem-16-871-s001.pdf]

BEILSTEIN JOURNAL OF ORGANIC CHEMISTRY

Supporting Information

for

A method to determine the correct photocatalyst concentration for photooxidation reactions conducted in continuous flow reactors

Clemens R. Horn and Sylvain Gremetz

Beilstein J. Org. Chem. **2020,** *16,* 871–879. doi:10.3762/bjoc.16.78

Descriptions of material and methods, flow setup, and LED characterization

License and Terms: This is a supporting information file under the terms of the Creative Commons Attribution License (http://creativecommons.org/licenses/by/4.0). Please note that the reuse, redistribution and reproduction in particular requires that the authors and source are credited.
The license is subject to the *Beilstein Journal of Organic Chemistry* terms and conditions: (https://www.beilstein-journals.org/bjoc)

Materials and methods

UV–vis spectra were recorded on an Agilent Cray 60 UV–vis spectrometer, using a Hellma High Precision Cell (light path 0.5 mm). All samples were collected in graduated flasks using a valve time-controlled by an Arduino microprocessor in order to validate the liquid flow rate. The conversion of alpha-terpinene was determined by NMR spectroscopy. The ^{13}C-decoupled ^{1}H NMR spectra were recorded with a Magritek Spinsolve 43 Carbon Ultra. The NMR samples were measured as collected. Citronellol samples were quenched with 2 equiv of a 0.25 N solution of PPh_3 in dichloromethane, and the conversion was determined by GC using tetradecane as an internal standard. The emission spectra of the LEDs were measured using a HERA Admesy spectroradiometer.

All solvents used were HPLC grade. The compounds were purchased and used as received from Sigma Aldrich (phenothiazine 98%, methylene blue, eosin Y 99%, erythrosin B 90%, fluorescein, citronellol 95%) and TCI (rose bengal, 9,10-dicyanoanthracene 98%, 9,10-dimethylanthracene 98%, tetraphenylporphyrin 98%, tris(2,2'-bipyridyl)ruthenium(II) chloride hexahydrate 98%, alpha-terpinene 90%).

Experimental procedure

As a flow reactor system, the commercially available Corning® Advanced-Flow™ Lab Photo Reactor was used (Figure S1). The solution containing the compound to be oxidized and the photosensitizer were pumped at a given flow rate (e.g., 1 N alpha-terpinene in toluene, flow rate 1 mL/min). The gas flow was started with the corresponding flow rate (here 44.8 mL/min at 1 bar), and the light source was switched on. The back pressure regulator was controlled using compressed nitrogen, and the pressure was increased until 8 bar, as measured at the entrance of the photoreactor. Samples were collected after a

liquid volume corresponding to at least 3 times the reactor volume had passed (here 3 × 2.7 mL at 1 mL/min = 8.1 min). Samples were collected into a graduated flask using a valve controlled with an Arduino [1]. The waste was collected in a bottle permanently purged with nitrogen gas.

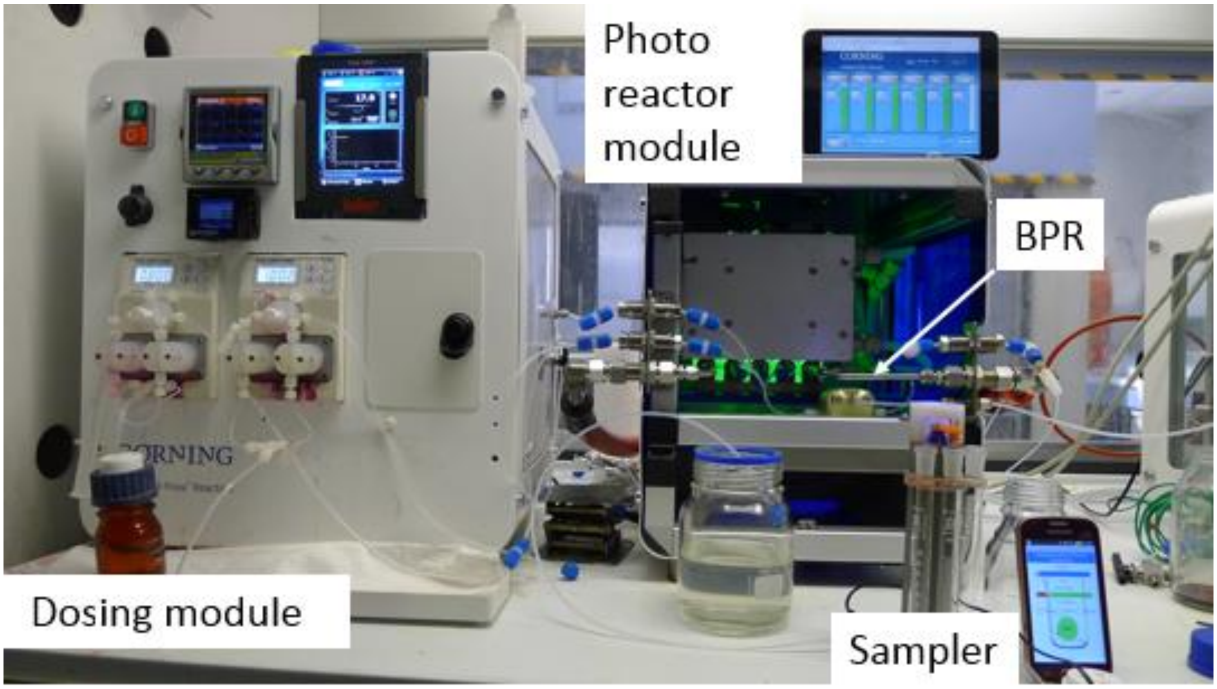

Figure S1: Corning® Advanced-Flow™ Lab Photo Reactor setup (the front protection was removed for the picture. Note: do not run a photochemical experiment with an open photoreactor).

Characterization of the light source

The distance of the light source to the fluidic module (3.4 cm) was fixed. This was optimized earlier using Zemax (www.zemax.com) in order to guarantee a homogenous illumination (Figure S2).

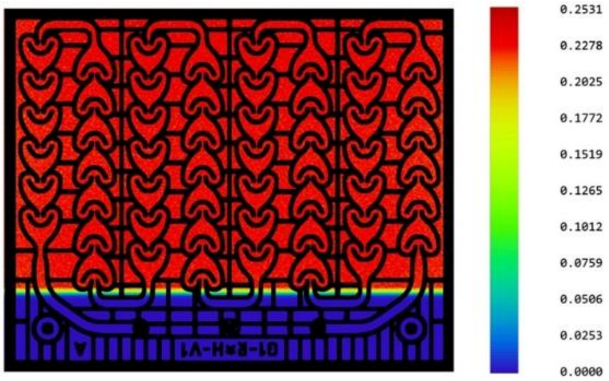

Figure S2: Calculated illumination at a 3.4 cm distance (LED-to-module).

Two LED panels containing 6 different wavelengths were used. The wavelength 344 nm was not used. The light power was measured using a spectroradiometer (HERA Admesy), and the measured spectra are shown in Figure S3.

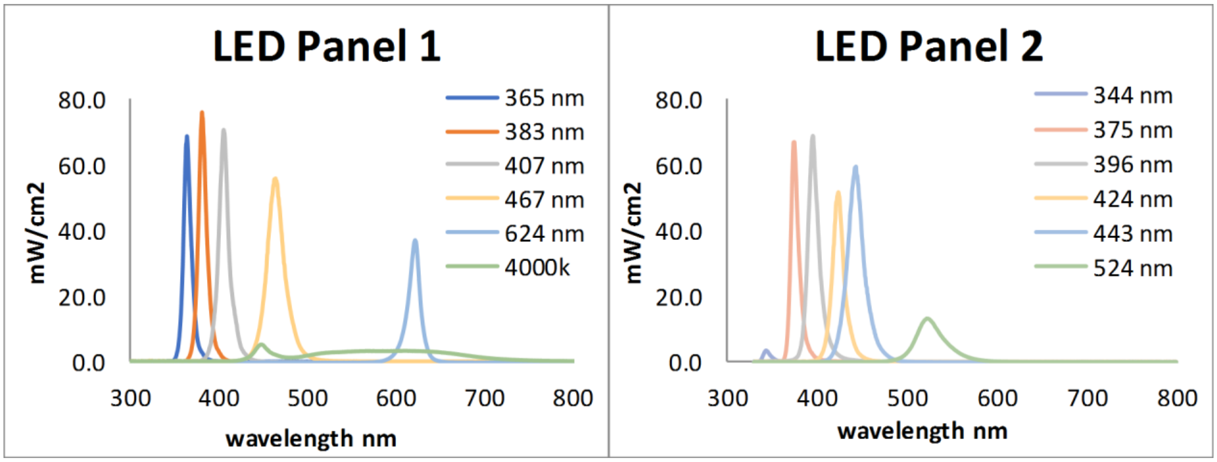

Figure S3: Emission spectra of the LEDs.

Analyses

^{1}H NMR analysis was prefered as the much faster method (1 min for one NMR mesurement vs 40 min for GC (triple injection)) [2]. Figure S4 shows three

representative ^{1}H NMR spectra of alpha-terpinene (1 N) in toluene. The top shows a starting solution while the middle and bottom show an incomplete reaction (3 mL/min flow rate). The top and middle spectra are ^{13}C-decoupled, and the bottom spectrum is a standard ^{1}H NMR spectrum. The integration of the double bond region of the terpene and using the signals of the aromatic system of toluene as references indicated 75% conversion vs 77% found by via GC.

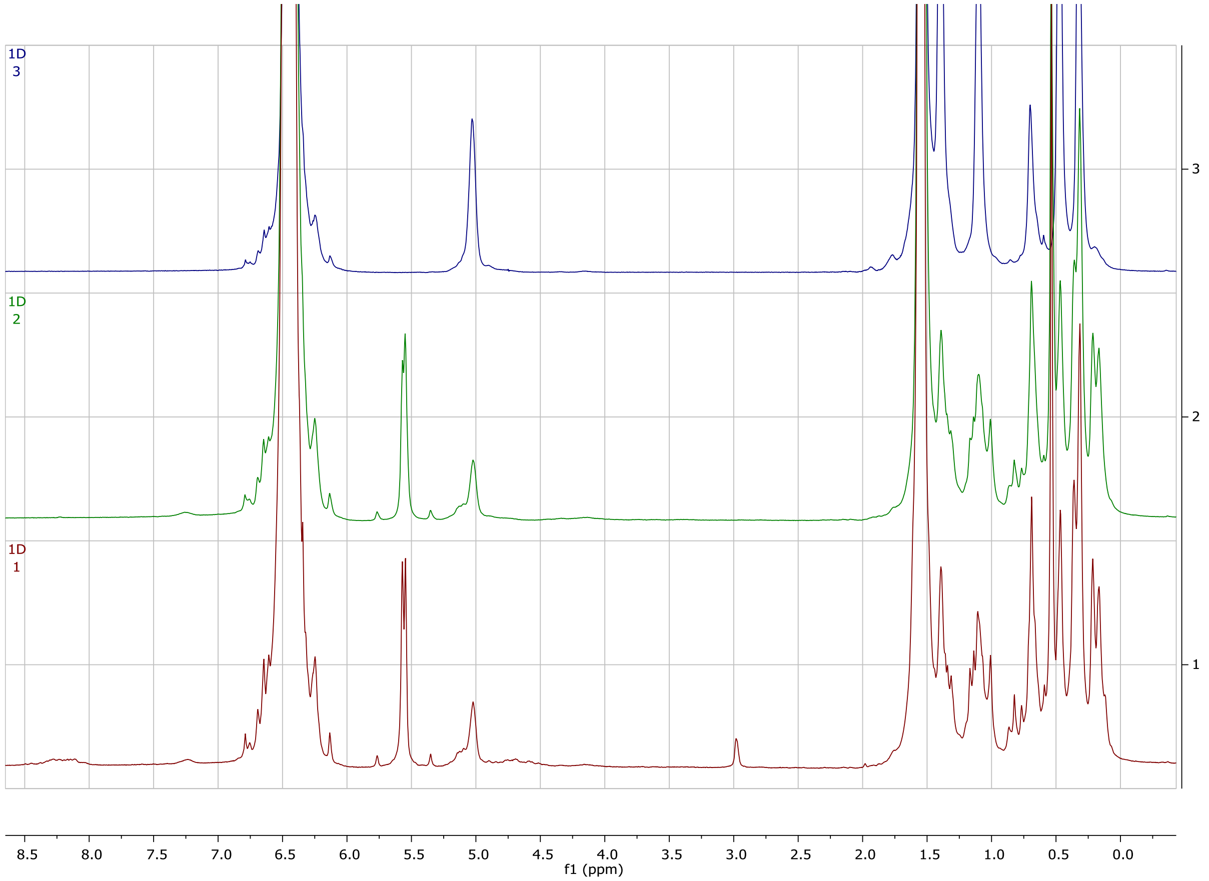

Figure S4: ^{13}C-decoupled ^{1}H NMR spectra of the starting solution (top), the incomplete reaction (middle), and a regular ^{1}H NMR spectrum of the incomplete reaction (bottom).

References

1. Details on the sampler: Material list, STL files for the printed parts and application for android phones can be requested from the author

2. Abdiaj, I.; Horn, C. R.; Alcazar, J. *J. Org. Chem.* **2019**, *84*, 4748-4753.
